# Impact of index hopping and bias towards the reference allele on accuracy of genotype calls from low-coverage sequencing

**DOI:** 10.1186/s12711-018-0436-4

**Published:** 2018-12-13

**Authors:** Roger Ros-Freixedes, Mara Battagin, Martin Johnsson, Gregor Gorjanc, Alan J. Mileham, Steve D. Rounsley, John M. Hickey

**Affiliations:** 1 The Roslin Institute and Royal (Dick) School of Veterinary Studies, The University of Edinburgh, Easter Bush, Midlothian, Scotland UK; 2 Department of Animal Breeding and Genetics, Swedish University of Agricultural Sciences, Box 7023, 750 07 Uppsala, Sweden; 3 Genus plc, 1525 River Road, DeForest, WI 53532 USA

## Abstract

**Background:**

Inherent sources of error and bias that affect the quality of sequence data include index hopping and bias towards the reference allele. The impact of these artefacts is likely greater for low-coverage data than for high-coverage data because low-coverage data has scant information and many standard tools for processing sequence data were designed for high-coverage data. With the proliferation of cost-effective low-coverage sequencing, there is a need to understand the impact of these errors and bias on resulting genotype calls from low-coverage sequencing.

**Results:**

We used a dataset of 26 pigs sequenced both at 2× with multiplexing and at 30× without multiplexing to show that index hopping and bias towards the reference allele due to alignment had little impact on genotype calls. However, pruning of alternative haplotypes supported by a number of reads below a predefined threshold, which is a default and desired step of some variant callers for removing potential sequencing errors in high-coverage data, introduced an unexpected bias towards the reference allele when applied to low-coverage sequence data. This bias reduced best-guess genotype concordance of low-coverage sequence data by 19.0 absolute percentage points.

**Conclusions:**

We propose a simple pipeline to correct the preferential bias towards the reference allele that can occur during variant discovery and we recommend that users of low-coverage sequence data be wary of unexpected biases that may be produced by bioinformatic tools that were designed for high-coverage sequence data.

## Background

Sequence data has the potential to empower identification of causal variants that underlie quantitative traits or diseases, to enhance livestock breeding, and to increase the precision and scope of population genetic studies. For sequence data to be used routinely in research and breeding, low-cost sequencing strategies must be used to assemble large datasets that cover most of the genetic diversity in a population. Such low-cost strategies could involve sequencing large numbers of individuals at low coverage, followed by imputation of whole-genome sequence data [1–3].

Current sequencing technologies have inherent sources of errors and bias that affect the quality of the resulting sequence data. Some biases affect the ability to successfully generate and align reads that cover regions with structural complexity, extreme base compositions, or particular sequence motifs [4–8]. Biases, together with other sources of errors, also increase the error rate in genotype calls [9–11]. Among these, two of the most important causes of incorrect genotype calls are index hopping and preferential bias of some bioinformatic tools towards the reference allele. The impact of these artefacts is likely greater for low-coverage data than for high-coverage data because low-coverage data has scant information and many standard tools for processing sequence data were designed for high-coverage data. With the proliferation of cost-effective low-coverage sequencing, there is a need to understand the impact of these artefacts on resulting genotype calls.

Index hopping has a biochemical cause and appears in the early stages of sequencing. Currently, the most widely used high-throughput sequencing platform is the HiSeq series of instruments from Illumina Inc. Due to the large sequencing capacity of these platforms, several samples are often sequenced jointly within a single flow cell channel by multiplexing. To link multiplexed sequence reads to the original samples, the adapter sequences used during library preparation include a set of unique index sequences. However, spurious extensions of library fragments with an incorrect sample index can occur during the exclusion-amplification (ExAmp) clustering that is used by the Illumina instruments when free adapters are present in a library pool. This leads to misassignment of sequence reads between samples in the multiplex. Recently, alarming data showed index hopping incidences of up to 10% [12]. These results sparked debate and concern about index hopping, although some subsequent studies reported a low incidence for most applications [13–15], which is in line with expectations if cleaning protocols are used to remove free adapters from the libraries [16]. While these results are reassuring, they pertained to high-coverage sequence data and the effect of index hopping on low-coverage sequence data and its downstream analysis remains unclear.

Preferential bias of some bioinformatic tools towards the reference allele (i.e., the allele that is present in the reference genome sequence) can be observed in sequence data following bioinformatic processing. It originates mainly during read alignment, but it can also occur during variant discovery and genotyping. Alignment of sequence reads onto a haploid reference genome relies on the calculation of similarity scores between reads and the reference genome. The more a read diverges from the reference, the less likely it is to align appropriately. This disfavours the alignment of reads that carry the alternative allele at a variant site because such reads have at least one additional mismatch to the reference genome compared to reads that carry the reference allele. If a read covers multiple variant sites and carries alternative alleles at multiple sites, the probability of aligning that read decreases even further, which in turn produces a stronger reference allele bias in highly polymorphic regions. This can lead to biases in downstream applications, e.g., in the estimation of allele frequencies [9, 17].

Another potential source of bias towards the reference allele can occur during variant discovery and genotyping. One of the most popular variant callers is GATK HaplotypeCaller [18], which provides a pipeline for efficient joint genotyping of multiple samples. In the GATK Best Practices pipeline, variant discovery and joint genotyping of multiple samples are performed as two separate steps [18, 19]. In the variant discovery step, read information for each site of the reference genome is stored for each individual sample in a gVCF file, which differs from the traditional VCF file in that it stores information of the non-variant sites as well as the variant sites. In the joint genotyping step, the gVCF files that have been created separately for each individual are combined, and genotypes are called for all individuals at all sites that are variant for at least one individual in the sequenced population. Compared to other pipelines, this two-step process has the advantage that only the genotyping is done jointly for all the samples and not the variant discovery itself, which is the most computationally demanding step. This two-step process improves scalability and facilitates incorporation of new batches of sequenced individuals for the joint genotyping step. However, GATK HaplotypeCaller was designed for high-coverage sequencing and, to our knowledge, its performance in low-coverage sequencing has not been assessed.

In this study, we explored the impact of index hopping and bias towards the reference allele in low-coverage sequence data. We show that index hopping and bias towards the reference allele due to alignment have little impact on genotype calls. However, unexpected biases may arise from pipelines that apply tools that were designed for high-coverage sequence data to low-coverage sequence data. In particular, we describe how a function from GATK HaplotypeCaller that is very useful for high-coverage data introduces a strong bias towards the reference allele when used on low-coverage data. We propose a new pipeline that avoids this bias. The results in this paper show the importance of validating the performance of tools designed for high-coverage data on low-coverage data.

## Methods

### Sequenced individuals

Twenty-six commercial pigs were used in this study. Tissue samples were collected from ear punches or tail clippings and genomic DNA was extracted using Qiagen DNeasy 96 Blood & Tissue kits (Qiagen Ltd., Mississauga, ON, Canada). Paired-end library preparation was conducted using the TruSeq DNA PCR-free protocol (Illumina, San Diego, CA). Two sets of libraries were produced; one with an average insert size of 350 bp and the other with an average insert size of 550 bp. Libraries with an average insert size of 350 bp were sequenced on a HiSeq 4000 instrument (Illumina, San Diego, CA), for a target coverage of 2× per sample. For this, all 26 samples were multiplexed within a single flow cell channel. Libraries with an average insert size of 550 bp were sequenced on a HiSeq X instrument (Illumina, San Diego, CA), for a target coverage of 30× per sample. For this, the 26 samples were sequenced, one sample per flow cell channel. All libraries were sequenced at Edinburgh Genomics (Edinburgh Genomics, University of Edinburgh, Edinburgh, UK). DNA samples from the same pigs were also genotyped using the GGP-Porcine HD BeadChip (GeneSeek, Lincoln, NE).

### Variant discovery

DNA sequence reads were pre-processed using Trimmomatic [20] to remove adapter sequences from the reads. Then, the reads were aligned to the reference genome *Sscrofa11.1* (GenBank accession: GCA_000003025.6) using the BWA-MEM algorithm with default settings and the option of marking shorter split hits as secondary alignment [21]. Duplicates were marked with Picard (http://broadinstitute.github.io/picard). Single nucleotide polymorphisms (SNPs) and short insertions and deletions (indels) were identified with the variant caller GATK HaplotypeCaller (GATK 3.8.0) [18, 19]. The GATK HaplotypeCaller performs local re-assembly of the reads to generate a list of possible haplotypes in a region by constructing a read-threading graph. Sections of that graph, which are supported by a number of reads (kmers) smaller than a predefined threshold, are considered to be likely sequencing errors and removed from the graph in a step referred to as ‘pruning’. By default, the threshold for pruning is set to ‘–minPruning 2’. We used the default settings but we also performed variant discovery without pruning (–minPruning 1). Variant discovery with GATK HaplotypeCaller was performed separately for each individual. A joint variant set for the 26 individuals was obtained by extracting the variant sites from all the individuals with GATK GenotypeGVCFs. Finally, biallelic SNPs were extracted with VCFtools [22]. To minimise computing costs, we considered variants on chromosome 1 only.

### Genotyping

We did not use genotypes that were called directly by GATK GenotypeGVCFs or any other software tool. Instead, we extracted allele read counts (i.e., the coverage that each allele received at each variant site) from the VCF file. Then, we called genotypes based on genotype probabilities that were calculated from allele read counts of the reference allele (nRef) and the alternative allele (nAlt). Genotype probabilities for the reference homozygote (0), heterozygote (1), and alternative homozygote (2) were calculated, respectively, as:

$$\begin{aligned} {\text{p}}\left( 0 \right) = \left( {1 - {\text{e}}} \right)^{\text{nRef}} \cdot{\text{e}}^{\text{nAlt}} , \hfill \\ {\text{p}}\left( 1 \right) = 0.5^{\text{nRef}} \cdot0.5^{\text{nAlt}} ,{\text{ and}} \hfill \\ {\text{p}}\left( 2 \right) = {\text{e}}^{\text{nRef}} \cdot\left( {1 - {\text{e}}} \right)^{\text{nAlt}} , \hfill \\ \end{aligned}$$

where *e* is the sequencing error rate, which was assumed to be 0.01. The three probabilities were scaled to sum to 1. Genotype calls were made at three levels of certainty: (1) the most probable genotypes (referred to as ‘best-guess’); (2) genotypes that had a probability greater than 0.90; or (3) genotypes that had a probability greater than 0.98.

### Genotype and allele concordance

Genotype concordance was calculated by: (1) comparing genotypes for the same variant from the sequence data and the SNP genotyping array, using the SNP array genotypes as being true; or (2) comparing the same variant from the sequence data at low and high coverage and using the high-coverage genotype calls as being true. Genotype concordance was calculated as the percentage of matches between the true genotypes and the genotype calls. We used the genotypes from all SNPs on chromosome 1 for which there was evidence of allele segregation based on the SNP genotyping array data of the 26 individuals and that were successfully discovered based on the sequence data of these individuals. The number of SNPs tested for concordance with SNP genotyping array data was equal to 5136 for the low-coverage data and 5531 for the high-coverage data. The same set of 5531 SNPs was also used to test the concordance between the low- and high-coverage sequence data. We also calculated allele concordance, as the percentage of matched alleles between the true genotypes and the genotype calls.

### Bias towards the reference allele due to variant caller and new pipeline

Initially, we called genotypes using the read counts stored in the gVCF files produced by GATK HaplotypeCaller. For testing potential biases introduced by the variant caller, we also called genotypes using the read counts that were obtained directly from the aligned reads stored in the BAM files. To do this, we extracted the read counts from the BAM files for the variant sites discovered by GATK HaplotypeCaller using pysam (version 0.13.0; https://github.com/pysam-developers/pysam), which is a wrapper around htslib and the samtools package [23]. We excluded reads with a mapping quality MAPQ lower than 20, reads that were not mapped in a proper pair, and secondary alignments. We refer to this method as the ‘new’ pipeline.

Our initial results indicated that there was a strong bias towards the reference allele introduced by the variant caller. Therefore, for all further analyses we used read counts that were obtained from the BAM files with the new pipeline for genotyping. We called genotypes for the 5531 variant sites on chromosome 1 discovered from the high-coverage sequence data that had already been genotyped using the SNP genotyping array.

### Bias towards the reference allele due to alignment

In this study, we defined alignment bias to be the differential alignment of almost-identical reads that differed only in one allele at a given variant site, regardless of whether it was the reference or alternative allele. To quantify the alignment bias, we aligned the 2× data against two reference genomes: the ‘original’ reference genome (GenBank accession: GCA_000003025.6) and a ‘tailored’ reference genome. The tailored reference genome was created by replacing the reference allele with the alternative allele at all variant sites discovered across the 26 individuals with the 30× sequence data from chromosome 1. Thus, the allele that was originally the alternative allele became the reference allele in the tailored reference genome and vice versa. We extracted the allele read counts from the aligned reads in the BAM files that were generated with both reference genomes. The allele read counts were used to call genotypes for evaluating genotype concordance between the 2× data and the true genotypes (from the 30× data). Genotypes were called from the allele read counts obtained with either: (1) the original reference genome (REF), or (2) the tailored reference genome (ALT). Because REF could favour the alignment of reads that carry the reference allele and disfavour the alignment of reads that carry the alternative allele, and vice versa for ALT, we also considered two additional cases that were a combination of the previous two: (3) read counts for the reference allele from the original reference genome and read counts for the alternative allele from the tailored reference genome (CIS), and (4) vice versa, read counts for the reference allele from the tailored reference genome and read counts for the alternative allele from the original reference genome (TRANS). Thus, the CIS case used allele read counts that had a more favourable alignment for each allele, and, in contrast, the TRANS case used allele read counts that had a more unfavourable alignment for each allele.

### Index hopping

In order to quantify the incidence of index hopping in our 2× dataset, we generated 2× data that either were free of index hopping or had different levels of simulated index hopping. The 2× data free of index hopping were generated by down-sampling the 30× data (i.e., random sampling of ~ 1/15 of the 30× reads), which had been generated without multiplexing (1 sample per lane). The resulting down-sampled 2× data was used to obtain baseline sequence data in the absence of index hopping. Then, index hopping was introduced in this data by randomly assigning reads to other individuals with a probability of 0.1, 0.5, 1, 2, or 5%. For each of these cases, we down-sampled the data independently before simulating index hopping to account for the random sampling of reads that occurs during sequencing.

To analyse the data, genotypes in each dataset were called as described above (best-guess or above a certain probability threshold) but also with an additional method that was more sensitive to index hopping based on the presence/absence of each allele. With this presence/absence method, the presence of a single read that supported the opposite allele was sufficient to change the genotype call (e.g., the genotype call with nRef = 10 and nAlt = 0 would be homozygous but the genotype call with nRef = 10 and nAlt = 1 would be heterozygous). Note that this method is equivalent to calling best-guess genotypes with null sequencing error rate.

To predict the level of index hopping level in the observed 2× dataset, we regressed the percentages of genotype concordance on the level of index hopping. Concordance percentages represent relative, rather than absolute, information and therefore should not be analysed using standard statistical techniques that are defined in real space, which has an absolute scale [24]. In order to validate the estimates of the level of index hopping with a methodology that was more appropriate for compositional data, we also analysed the data using isometric log-ratio transformations (ilr) of the concordance percentages [25, 26]. The ilr were the log-ratios of the percentage of correct calls against the percentages of incorrect calls or the log-ratios of the percentage of correct homozygous calls against the percentage of incorrect heterozygous calls. We fitted a quadratic regression of the ilr variables on the level of index hopping.

## Results

### Variant discovery

Most of the SNPs present on the SNP genotyping array were discovered using sequence data, both at high and low coverage. The number of biallelic SNPs discovered on chromosome 1 with high- and low-coverage data is in Table 1. A total of 1,693,308 biallelic SNPs were discovered with the high-coverage data and 1,333,943 with the low-coverage data. The low-coverage sequence data contained 76.3% (1,292,269) of the biallelic SNPs that were discovered with the high-coverage data. The SNP genotyping array had 5779 SNPs on chromosome 1 that segregated in the 26 sequenced individuals. Of these, 95.7% (5531) were discovered with the high-coverage data and 88.9% (5136) with the low-coverage data.

**Table 1:** Number of biallelic SNPs discovered on chromosome 1 with low and high sequencing coverage and percentage of overlap with the SNP genotyping array

	Low coverage	High coverage
Number of variants	1,333,943	1,693,308
Overlap with high-coverage data	96.9%	–
Overlap with low-coverage data	–	76.3%
Overlap with the SNP genotyping array^a^	88.9%	95.7%

^a^Relative to the 5779 variants present in the SNP genotyping array GGP-Porcine HD BeadChip (GeneSeek, Lincoln, NE) that segregated in the 26 individuals tested

Disabling the pruning step in GATK HaplotypeCaller for processing the low-coverage data increased the number of variants discovered but also the number of potential false positives. The numbers of biallelic SNPs discovered on chromosome 1 with low-coverage data with or without pruning are in Table 2. When pruning was disabled, 1,877,644 biallelic SNPs were discovered with the low-coverage data. This number was larger than the set of variants discovered with the high-coverage data with the default pruning settings (Table 1). However, 24.1% of these extra SNPs could not be validated using the high-coverage data, which is a much greater proportion than when pruning was used (3.1%).

**Table 2:** Number of biallelic SNPs discovered on chromosome 1 with low sequencing coverage with different GATK HaplotypeCaller pruning options, the percentage of variants not validated with high sequencing coverage, and genotype and allele concordances with the SNP genotyping array

	minPruning = 2 (default)	minPruning = 1
Number of variants	1,333,943	1,877,644
Not validated at high coverage	3.1%	24.1%
Best-guess genotype concordance	62.1%	76.5%
Allele concordance	77.6%	87.5%

### Genotype concordance and bias towards the reference allele due to variant caller

The variant caller that we used introduced a bias towards the reference allele and this had a large impact on genotype calling with low-coverage data. Table 3 shows the genotype concordance for calls that were obtained with the allele read counts from the gVCF files produced by GATK HaplotypeCaller. This table shows that there was a large bias towards the reference allele with the low-coverage sequence data. In the most extreme case of sites with 1× coverage, we would expect the genotypes that are heterozygous according to the SNP genotyping array to be called as either of the two possible homozygotes, ‘0’ and ‘2’, 50% of the times. Instead, we called them as reference homozygotes ‘0’ 95.1% of the times and as alternative homozygotes ‘2’ only 4.9% of the times. Also, at 1× coverage, 82.0% of the alternative homozygotes ‘2’ were called as reference homozygotes ‘0’. Because of this bias, the overall genotype concordance was only 62.1% and the allele concordance was only 77.6%.

**Table 3:** Concordance of best-guess genotype calls from sequence data with SNP array genotypes, using allele read counts obtained with the default settings of GATK HaplotypeCaller

	n^a^	Genotype concordance (%)	Allele concordance (%)	Concordance by genotype (%)								
				True = 0			True = 1			True = 2		
				0|0	1|0	2|0	0|1	1|1	2|1	0|2	1|2	2|2
Low coverage												
1×	27,185	42.2	61.0	99.97	–	0.03	95.14	–	4.86	81.96	–	18.04
2×	33,638	57.2	76.0	99.94	0.00	0.06	72.87	3.51	23.62	20.07	0.25	79.68
3×	24,789	70.3	84.5	99.91	0.08	0.01	56.37	31.87	11.76	6.23	1.45	92.32
4×	14,015	79.7	89.6	99.85	0.13	0.02	43.11	51.44	5.46	2.14	1.69	96.16
5×	6502	85.6	92.7	99.93	0.04	0.04	32.65	64.75	2.59	0.90	1.96	97.14
6–10×	3705	90.5	95.2	99.83	0.12	0.06	22.47	74.68	2.85	0.61	1.07	98.32
Overall	109,834	62.1	77.6	99.92	0.04	0.03	66.41	21.50	12.09	29.84	0.71	69.45
High coverage	131,806	99.7	99.9	99.80	0.19	0.01	0.21	99.72	0.07	0.17	0.16	99.68

^a^Number of genotypes called across 26 individuals at 5136 and 5531 SNPs for low- and high-coverage data, respectively

Concordance is shown by coverage at variant site

The bias towards the reference allele due to the variant caller can be avoided by calling genotypes from the read counts that are obtained directly from the aligned reads stored in BAM files. Table 4 shows the genotype concordance obtained with the new pipeline using allele read counts that were extracted directly from BAM files. The bias was corrected and the concordances matched expectations. Overall, genotype and allele concordances rose to 81.1 and 90.5%, respectively. As expected, most of the incorrect calls arose from the difficulty of calling heterozygous genotypes at low coverage.

**Table 4:** Concordance of best-guess genotype calls from sequence data with SNP array genotypes, using allele read counts obtained from aligned reads in BAM files

	n^a^	Genotype concordance (%)	Allele concordance (%)	Concordance by genotype (%)								
				True = 0			True = 1			True = 2		
				0|0	1|0	2|0	0|1	1|1	2|1	0|2	1|2	2|2
Low coverage												
1×	28,300	62.1	80.8	99.34	–	0.66	51.46	–	48.54	0.96	–	99.04
2×	32,699	79.5	89.7	98.42	1.53	0.05	26.41	48.15	25.44	0.21	1.70	98.09
3×	25,993	88.3	94.1	98.25	1.72	0.03	14.01	71.98	14.01	0.12	2.36	97.52
4×	16,346	92.5	96.3	97.91	2.09	0.00	8.36	83.84	7.80	0.00	2.77	97.23
5×	8878	94.9	97.5	97.28	2.72	0.00	4.83	91.15	4.02	0.16	2.81	97.03
6–10×	6444	95.0	97.5	97.43	2.50	0.07	5.01	91.09	3.90	0.00	2.75	97.25
Overall	118,660	81.1	90.5	98.39	1.43	0.18	24.43	52.30	23.27	0.33	1.71	97.96
High coverage	131,782	99.8	99.9	99.80	0.19	0.01	0.12	99.81	0.07	0.10	0.17	99.73

^a^Number of genotypes called for 5531 SNPs across 26 individuals both for low- and high-coverage data

Concordance is shown by coverage at variant site

Disabling pruning was not as good a solution for correcting the bias as the new pipeline of extracting the allele read counts from the BAM files. Table 2 shows genotype and allele concordances with the default pruning setting and without pruning. Without pruning, the genotype and allele concordances rose to 76.5 and 87.5%, respectively, but these percentages were lower than with the new pipeline.

Once the bias towards the reference allele due to the variant caller was corrected, the concordance at homozygous sites was very high, regardless of the conservativeness of the genotype calls, but these thresholds were important for concordance at heterozygous sites. Table 5 shows genotype concordance between calls with low- and high-coverage data obtained as best-guess genotypes or with a minimum probability of 0.90 or 0.98. At reference and alternative homozygous sites, the best-guess genotypes had an overall concordance of 98.5 and 98.2%, which was greater than the concordance of calls with a minimum probability of 0.90 (97.2 and 96.4%, respectively), despite the latter being called with a greater level of certainty. The reason for this is that with a minimum probability of 0.90, there is not enough certainty for calling any genotype at sites with a coverage of 1×, and at sites with a coverage of 2× or 3×, only potential heterozygotes (either true or false), but not homozygotes, can be called due to the considered error rate. While the number of homozygotes that were incorrectly called as heterozygous was actually very small, the impact of these incorrect calls on overall concordance was noticeable because the low-coverage data had many more loci with 2× and 3× coverage than with 4× or greater coverage. A similar situation occurred with genotype calls that had a minimum probability of 0.98.

**Table 5:** Concordance between genotype calls with different levels of conservativeness from low- and high-coverage sequence data, using allele read counts obtained from aligned reads in BAM files

	n^a^	Genotype concordance (%)	Allele concordance (%)	Concordance by genotype (%)									
				True = 0			True = 1			True = 2			
				0|0^b^	1|0	2|0	0|1	1|1	2|1	0|2		1|2	2|2
Best-guess													
1×	30,875	62.6	81.1	99.45	–	0.55	51.35	–	48.65	0.70		–	99.30
2×	35,688	79.9	90.0	98.60	1.40	0.00	26.20	48.26	25.54	0.04		1.61	98.35
3×	28,357	88.6	94.3	98.36	1.64	0.00	13.80	72.20	14.00	0.00		2.22	97.78
4×	17,849	92.7	96.4	98.05	1.95	0.00	8.22	84.01	7.77	0.00		2.63	97.37
5×	9619	95.3	97.6	97.40	2.60	0.00	4.55	91.52	3.93	0.00		2.49	97.51
6–10×	7047	95.2	97.6	97.73	2.27	0.00	4.96	91.05	4.00	0.00		2.68	97.32
Overall	129,435	81.4	90.7	98.53	1.34	0.13	24.27	52.40	23.33	0.18		1.61	98.21
Probability ≥ 0.90													
1×	0	–	–	–	–	–	–	–	–	–		–	–
2–3×^b^	14,572	95.5	97.7	–	100.00	–	–	100.00	–	–		100.00	–
4×	14,359	92.7	96.3	99.97	0.03	0.00	16.24	68.39	15.37	0.00		0.05	99.95
5×	8315	96.3	98.2	99.92	0.08	0.00	6.76	87.41	5.83	0.00		0.10	99.90
6–10×	6397	98.1	99.0	99.83	0.17	0.00	3.08	94.65	2.27	0.00		0.29	99.71
Overall	43,643	95.1	97.5	97.18	2.82	0.00	3.52	93.26	3.21	0.00		3.65	96.35
Probability ≥ 0.98													
1–3×	0	–	–	–	–	–	–	–	–	–		–	–
4–5×^b^	4366	99.8	99.9	–	100.00	–	–	100.00	–	–		100.00	–
6–10×	6313	98.1	99.1	99.83	0.17	0.00	3.15	94.58	2.26	0.00		0.29	99.71
Overall	10,679	98.8	99.4	99.65	0.35	0.00	1.00	98.28	0.72	0.00		0.57	99.43

^a^Number of genotypes called for 5,531 SNPs across 26 individuals

^b^Heterozygotes are easier to call than homozygotes; at these coverages, certainty is not sufficient to call the homozygotes, but note, that the actual counts for (1|0) and (1|2) are very low compared to (1|1): 19-fold and 569-fold lower for genotypes called with a probability greater than 0.90 and 0.98, respectively

Concordance is shown by coverage at variant site

At heterozygous loci, it was very difficult to call heterozygotes at the lowest coverages. Because of the large proportion of loci with low coverage, the genotype concordance of heterozygous loci with best-guess genotypes was 52.4%. With more conservative calls, the heterozygotes were called more accurately and the genotype concordance was 93.3 and 98.3% with minimum probabilities of 0.90 and 0.98, respectively. However, there was a trade-off between the concordance of called genotypes and the number of called genotypes. With more conservative calls, the number of called genotypes was only a small fraction of those that could be called using best-guess genotypes: 33.7% with a minimum probability of 0.90 and only 8.3% with a minimum probability of 0.98.

### Bias towards reference allele due to alignment

Reads with an allele that was present in the reference genome had a greater probability of successful alignment, but the difference was small. Table 6 shows the average allele read counts depending on which allele was in the reference genome. Approximately 1.3% of reads were not aligned when the reference genome contained the opposite allele than the read. The number of reads that carried the allele in the reference genome but that were incorrectly mapped to a site where the individual was homozygous for the opposite allele increased by 9.8% due to the alignment bias, but these potentially mismapped reads represented only a small fraction of the total.

**Table 6:** Average allele read counts depending on which allele is in the reference genome

Allele^a^	Reference genome	Allele in reference genome^a^	Overall	True genotype		
				0	1	2
Reference	Original	Reference	1.483	2.470	1.237	0.017
	Tailored^b^	Alternative	1.463	2.440	1.219	0.016
		*Difference not aligned* ^c^	*1.3%*	*1.2%*	*1.5%*	*9.8%*
Alternative	Original	Reference	0.980	0.014	1.217	2.407
	Tailored^b^	Alternative	0.993	0.016	1.234	2.438
		*Difference not aligned* ^c^	*1.3%*	*9.8%*	*1.3%*	*1.3%*

^a^Alleles are defined as reference or alternative allele based on the original pig reference genome *Sscrofa11.1* (GenBank assembly accession: GCA_000003025.6)

^b^The tailored reference genome was created by replacing the reference allele with the alternative allele at all variant sites discovered across the 26 individuals with the 30× sequence data from chromosome 1

^c^Proportion of reads that did not align when the reference genome carried the opposite allele

However, the impact of the bias towards the reference allele due to alignment on the genotype calls is likely to be small. Table 7 shows the genotype concordance between low- and high-coverage sequence data after alignment with the original reference genome (REF), the tailored reference genome (ALT), or a combination of both (CIS and TRANS). Use of the REF or ALT reference genomes introduced some bias towards homozygote calls for the reference or the alternative allele, respectively. Use of the CIS combination, where the allele read counts were obtained from the most favourable case for each (i.e., the reference genome contained that same allele), increased the number of genotype calls regardless of the conservativeness of the calls and increased the ability of correctly calling heterozygotes with lower levels of certainty. In contrast, use of the TRANS combination, where the allele read counts were obtained from the least favourable case for each (i.e., the reference genome contained the opposite allele), reduced the number of genotype calls and the ability to correctly call heterozygotes. Overall, changes in best-guess genotype concordance were small and the percentage of incorrect calls between the use of CIS (most favourable case) and REF (current practice) differed only by 0.1 absolute percentage points.

**Table 7:** Impact of bias towards the reference allele due to alignment on concordance between low- and high-coverage sequence data by alignment with the original reference genome (REF), the tailored reference genome (ALT), or a combination of both (CIS and TRANS)

	n^a^	Genotype concordance (%)	Allele concordance (%)	Concordance by genotype (%)								
				True = 0			True = 1			True = 2		
				0|0	1|0	2|0	0|1	1|1	2|1	0|2	1|2	2|2
Best-guess												
REF	129,435	81.4	90.7	98.53	1.34	0.13	24.27	52.40	23.33	0.18	1.61	98.21
ALT	129,327	81.4	90.7	98.36	1.49	0.14	23.66	52.42	23.92	0.17	1.47	98.36
CIS	129,610	81.5	90.7	98.37	1.49	0.14	23.82	52.73	23.45	0.17	1.62	98.21
TRANS	129,148	81.3	90.6	98.52	1.34	0.13	24.11	52.10	23.79	0.18	1.46	98.36
Probability ≥ 0.90												
REF	43,643	95.1	97.5	97.18	2.82	0.00	3.52	93.26	3.21	0.00	3.65	96.35
ALT	43,489	95.0	97.5	96.75	3.25	0.00	3.35	93.30	3.36	0.00	3.22	96.78
CIS	43,970	95.0	97.5	96.88	3.12	0.00	3.44	93.30	3.26	0.00	3.52	96.48
TRANS	43,145	95.1	97.6	97.10	2.90	0.00	3.42	93.28	3.30	0.00	3.32	96.68
Probability ≥ 0.98												
REF	10,679	98.8	99.4	99.65	0.35	0.00	1.00	98.28	0.72	0.00	0.57	99.43
ALT	10,638	98.8	99.4	99.64	0.36	0.00	0.92	98.26	0.81	0.00	0.41	99.59
CIS	10,858	98.8	99.4	99.65	0.35	0.00	0.98	98.23	0.78	0.00	0.55	99.45
TRANS	10,463	98.8	99.4	99.64	0.36	0.00	0.94	98.31	0.75	0.00	0.43	99.57

^a^Number of genotypes called for 5531 SNPs across 26 individuals

### Index hopping

Index hopping was estimated to be around 1.5% in our dataset. The results of using the method based on presence/absence of each allele, which is more sensitive to index hopping, are in Table 8, which shows the genotype concordance for the real and simulated data. Regression of genotype concordance for homozygotes on the level of index hopping had a very high R^2^ (≥ 0.99), while the R^2^ was less than 0.05 for heterozygotes. Similarly, regression of ilr transformations of concordance on the level of index hopping also had a high R^2^ when calculated for homozygotes (≥ 0.98). In all cases, the level of index hopping was estimated to range from 1.3 to 1.8%.

**Table 8:** Estimates of index hopping incidence through concordance between low- and high-coverage sequence data in the real and simulated datasets, expressed as percentages and as isometric log-ratios

	Concordance by genotype (%)									Isometric log-ratios				
	True = 0			True = 1			True = 2			3 parts^a^			2 parts^b^	
	0|0	1|0	2|0	0|1	1|1	2|1	0|2	1|2	2|2	0|0 vs. 1|0, 2|0	1|1 vs. 0|1, 2|1	2|2 vs. 0|2, 1|2	0|0 vs. 1|0	2|2 vs. 1|2
Observed	98.45	1.42	0.13	24.15	52.62	23.23	0.18	1.71	98.10	4.44	0.65	4.22	3.00	2.86
Simulated														
0%	99.62	0.35	0.03	23.57	52.98	23.45	0.04	0.47	99.48	5.61	0.66	5.35	4.00	3.78
0.1%	99.53	0.44	0.03	23.59	53.52	22.89	0.08	0.52	99.40	5.47	0.68	5.06	3.83	3.72
0.5%	99.28	0.66	0.06	23.91	53.22	22.87	0.10	0.92	98.98	5.07	0.67	4.72	3.55	3.31
1%	98.99	0.90	0.10	23.70	53.23	23.07	0.14	1.33	98.53	4.72	0.67	4.43	3.32	3.04
2%	98.20	1.64	0.16	23.73	52.90	23.37	0.23	2.16	97.62	4.29	0.66	4.04	2.89	2.70
5%	96.34	3.29	0.37	23.56	53.37	23.07	0.59	4.75	94.66	3.65	0.68	3.29	2.39	2.12
Regression														
R^2^	0.999	0.998	0.999	0.014	0.044	0.014	0.989	1.000	0.999	0.993	0.213	0.981	0.995	0.989
Estimate	1.74	1.77	1.47	–	–	–	1.28	1.45	1.43	1.58	–	1.48	1.67	1.46

^a^The 3-part isometric log-ratios take the form $\sqrt {\frac{2}{3}} \ln \frac{(0|0)}{{\sqrt {\left( {1|0} \right)\cdot\left( {2|0} \right)} }}$

^b^The 2-part isometric log-ratios take the form $\frac{1}{\sqrt 2 }\ln \frac{(0|0)}{{\left( {1|0} \right)}}$

Results obtained using the concordance variables of best-guess genotypes and genotypes called with probabilities higher than 0.90 and 0.98, largely supported the results for the presence/absence calling method (data not provided). Results obtained using the concordance variables of best-guess genotypes gave estimates of the level of index hopping ranging from 1.3 to 1.8% (R^2^ ≥ 0.99). The concordance variables of genotypes with probabilities higher than 0.98 were less sensitive to index hopping and resulted in a lower regression fit and lower or unreliable estimates of the level of index hopping (1.1–1.3%, R^2^ = 0.96–0.99, for percentages; 1.4–1.7% but R^2^ = 0.81 to 0.97 for ilr). The concordance variables of the genotypes with probabilities higher than 0.90 were in between, with estimates ranging from 1.3 to 1.5% (R^2^ ≥ 0.99).

Table 9 shows the impact of different levels of index hopping on genotype concordance. Incidences of 1 or 2% of index hopping increased the percentage of incorrect calls from 17.8 to 18.1 or 18.7%, respectively, for best-guess genotypes, from 3.1 to 3.8 or 4.6%, respectively, for genotypes with a probability above 0.90, and from 0.6 to 0.8 or 0.9%, respectively, for genotypes with a probability above 0.98.

**Table 9:** Impact of level of index hopping on concordance between low- and high-coverage sequence data

	Genotype concordance (%)	Allele concordance (%)	Concordance by genotype (%)								
			True = 0			True = 1			True = 2		
			0|0	1|0	2|0	0|1	1|1	2|1	0|2	1|2	2|2
Best-guess											
0%	82.2	91.1	99.63	0.34	0.03	23.65	52.82	23.53	0.04	0.47	99.49
0.1%	82.4	91.2	99.55	0.41	0.03	23.67	53.36	22.97	0.08	0.49	99.43
0.5%	82.2	91.1	99.30	0.64	0.06	23.97	53.08	22.96	0.10	0.89	99.01
1%	81.9	90.9	99.03	0.87	0.10	23.77	53.09	23.14	0.14	1.28	98.58
2%	81.3	90.6	98.25	1.59	0.16	23.79	52.78	23.43	0.23	2.07	97.70
5%	80.1	89.9	96.45	3.17	0.37	23.63	53.23	23.14	0.59	4.58	94.82
Probability ≥ 0.90											
0%	96.9	98.5	99.20	0.80	0.00	2.46	94.98	2.55	0.00	1.16	98.84
0.1%	96.7	98.3	99.01	0.99	0.00	2.59	94.70	2.71	0.00	1.26	98.74
0.5%	96.4	98.2	98.39	1.61	0.00	2.59	94.75	2.66	0.00	1.98	98.02
1%	96.2	98.1	97.87	2.13	0.00	2.68	94.99	2.33	0.00	2.98	97.02
2%	95.4	97.7	96.36	3.64	0.00	2.59	94.85	2.56	0.00	4.92	95.08
5%	93.2	96.6	92.53	7.47	0.00	2.65	94.78	2.57	0.00	10.55	89.45
Probability ≥ 0.98											
0%	99.4	99.7	100.00	0.00	0.00	0.53	99.03	0.44	0.00	0.00	100.00
0.1%	99.3	99.6	100.00	0.00	0.00	0.63	98.85	0.53	0.00	0.00	100.00
0.5%	99.4	99.7	99.95	0.05	0.00	0.37	99.09	0.54	0.00	0.09	99.91
1%	99.2	99.6	99.82	0.18	0.00	0.51	98.97	0.52	0.00	0.46	99.54
2%	99.1	99.5	99.47	0.53	0.00	0.52	98.89	0.59	0.00	0.75	99.25
5%	98.6	99.3	98.42	1.58	0.00	0.59	98.94	0.47	0.00	2.83	97.17

## Discussion

We quantified the impact of different sources of sequencing errors and biases towards the reference allele on genotype calls derived from low-coverage data. Index hopping and bias towards the reference allele due to alignment had little impact on genotype calls. However, we found that variant callers can introduce a strong bias towards the reference allele and this has a large impact on genotype calls. This bias is likely to be pipeline specific [11], but we have detected it using one of the most popular tools for variant discovery. The step that causes this bias was designed for the processing of high-coverage data but introduces a systematic bias when it is applied to low-coverage data. Other unexpected biases may appear when tools designed for use with high-coverage data are used to process low-coverage data. Awareness of these biases allowed us to design a pipeline that gave significantly more accurate genotype calls from low-coverage sequence data than a standard pipeline. In the following, we discuss each of the sources of errors and biases that we have analysed and our proposed new pipeline for variant discovery and joint genotyping, which addresses the most important source of bias.

### Bias towards the reference allele due to variant caller

Tools that are designed for high-coverage sequence data can introduce unexpected biases when used to process low-coverage sequence data. We found that this was the case for the ‘pruning’ step implemented in GATK HaplotypeCaller. During variant discovery, it is virtually impossible to distinguish between a sequencing error and a genuine variant. In order to make variant discovery more robust, different tools use different strategies to identify potential sequencing errors. In the case of GATK HaplotypeCaller, this strategy is the ‘pruning’ step. GATK HaplotypeCaller performs local re-assembly of the reads to generate a list of possible haplotypes in a region by constructing a read-threading graph. Paths of this graph that are supported by a number of reads (kmers) equal or smaller than a predefined threshold are considered to be probably sequencing errors and are removed from the graph (pruned). In the next step of the HaplotypeCaller method, each individual read is aligned against each possible haplotype, including the reference, and a likelihood score is calculated for each read-haplotype pair. Then, the likelihood that a read carries each of the alleles at a site is calculated as the product of the likelihoods of all haplotypes that carry that allele. Finally, the allele with the greatest marginal likelihood is called.

While this is a reasonable strategy for high-coverage sequence data, it introduces a huge bias towards the reference allele when used for low-coverage sequence data. This can be understood intuitively with a simple example. Imagine that at any given site with the reference allele ‘A’ and the alternative allele ‘B’, we have only one read and that this read carries the alternative allele B. The graph path representing the haplotype with the allele B will be supported by only one read and will be pruned out of the graph with the default settings, where at least two reads supporting a path are required. This means that the only haplotype that remains in the graph path is the reference haplotype with allele A. Then, in the next step, this same read with allele B will be paired with all the possible haplotypes. In this case, the only possibility is the reference haplotype with allele A and therefore that read is called as carrying the reference allele A. Thus, instead of the true state with nRef = 0 and nAlt = 1, we end up with the opposite situation with nRef = 1 and nAlt = 0. The same bias would arise with a coverage of 3×, if two reads carry allele A and one read carries allele B. In that case, instead of the true state with nRef = 2 and nAlt = 1, which indicates a heterozygote, we end up with nRef = 3 and nAlt = 0, which indicates a reference homozygote. Then, these biased allele read counts are stored in a gVCF, the file that includes both the variant and non-variant sites and that is used for multi-sample joint genotyping.

The bias in our low-coverage data was so pervasive that it was carried over to downstream analyses and affected imputation accuracy at the population level. We performed preliminary analyses of whole-genome imputation using the hybrid peeling algorithm implemented in AlphaPeel [27] with sequence data of 1146 individuals, mostly with 2× coverage. We used a leave-one-out design to assess imputation accuracy on 84 individuals that were sequenced at high coverage. To test the impact of the bias on imputation accuracy, we used as input information either the biased allele read counts obtained with pruning or the non-biased allele read counts that were extracted directly from the aligned reads. We estimated that the individual-wise dosage correlations decreased by an average of 0.10 (0.04 SD; max. 0.20) and the individual-wise percentage of correct best-guess genotypes by 7.5 absolute percentage points (3.8% SD; max. 14.7%) as a result of this bias (unpublished data). The imputation algorithm that we used for this test accounts for uncertainty by calculating genotype probabilities from the allele read counts [27], but the impact of the bias on imputation accuracy could be even greater for imputation algorithms that instead take genotype calls as an input.

#### New pipeline

Based on our findings, we propose a new pipeline for variant discovery and genotype calling with low-coverage sequence data that takes advantage of the robustness provided by the pruning option of GATK HaplotypeCaller while avoiding bias towards the reference allele. The proposed pipeline has two steps: (1) variant discovery with the default pruning setting of GATK HaplotypeCaller; and (2) genotype calling from the aligned reads stored in the BAM files for the variants discovered.

##### Variant discovery with GATK HaplotypeCaller

In step 1 of the proposed pipeline, variant discovery is performed with GATK HaplotypeCaller with the default pruning setting on a per-individual basis. Disabling pruning does not seem an appropriate solution for variant discovery with low-coverage sequencing because this increases the number of potential false positives (Table 2), as well as computational time. The pruning option of GATK HaplotypeCaller makes variant discovery more robust to false positives, but there is a trade-off between specificity and sensitivity. While pruning reduces the ability to discover variants from low-coverage data, this can be overcome by sequencing strategies that target haplotypes from the population instead of individuals (e.g., AlphaSeqOpt; [28, 29]) in two ways: (1) sequencing individuals that share large amounts of haplotypes with the population at high coverage ensures discovery of many common variants [30]; and (2) given that the realized coverage at a base site follows a Poisson distribution and, therefore, every individual has a greater coverage than the average target coverage at many random sites, many variants can be discovered if a sufficiently large number of individuals are sequenced at low coverage, even if pruning is enabled. For instance, with only 26 individuals sequenced at 2× coverage, we discovered 76.3% of the variants that were discovered with the same individuals at 30× coverage. The gap between variants discovered at low or high coverage is expected to decrease with increasing sample sizes.

##### Genotype calling from aligned reads

In step 2 of the proposed pipeline, a joint list of variant sites is extracted from the individual VCF files and the allele read counts at these sites are extracted from the aligned reads stored in the BAM files for each individual. GATK HaplotypeCaller with pruning induces a bias towards the reference allele when used with low-coverage data. This bias is introduced during variant discovery but manifests itself in the genotype calls if the joint genotyping uses the allele read counts stored in the gVCF or VCF files that are produced by the variant caller. This bias can be avoided if we call genotypes based on allele read counts, which are extracted directly from the aligned reads that are stored in the BAM files using tools such as pysam (https://github.com/pysam-developers/pysam).

The proposed pipeline provides the scalability needed for routine incorporation of new batches of sequenced individuals, using a similar logic as the GATK Best Practices pipeline. In the latter pipeline, information for both variant and non-variant sites is stored for each individual in the gVCF files, which is used later for joint genotyping at all variant sites, but these gVCF or VCF files contain biased allele read counts for low-coverage data. In the new pipeline that we propose, we produce regular VCF files only to obtain a list of all variant sites that have been discovered across the sequenced samples, followed by extracting the raw allele read counts at those sites for all individuals. Using this pipeline, it is very easy to add new batches of samples without having to repeat the joint genotyping by simply extracting the allele read counts for the new individuals and the new variants discovered and adding them to any pre-existing dataset. This also reduces data storage needs because the VCF files are much smaller than the gVCF files.

The proposed pipeline simplifies the processing of large numbers of individuals that are sequenced at low coverage by using available tools. This pipeline gave better genotype and allele concordances than using GATK HaplotypeCaller with disabled pruning. Alternative pipelines based on tools such as SAMtools [23] or ANGSD [31] may be equally well-suited for low-coverage sequence data. Pipelines based on imputation tools such as STITCH [32] may also be unaffected by the bias introduced by the pruning step because, similar to the proposed pipeline, they directly exploit the information from the aligned reads (in this case, the phase information of the reads).

### Bias towards the reference allele due to alignment

With the current pig reference genome *Sscrofa11.1*, bias towards the reference allele due to alignment was very low and its impact on genotype calls was negligible. Our estimates suggest that 1.3% of the reads did not align because the reference genome contained the opposite allele to the read allele and this increased the percentage of incorrect best-guess genotype calls by only 0.1 absolute percentage points. The reference genome *Sscrofa11.1* was largely constructed using Pacific Biosciences long reads, with a coverage of 65× and provides much better mapping quality than the previous version *Sscrofa10.2* (GenBank accession: GCA_000003025.4). For example, in a 2× coverage sample, the percentage of mapped reads increased from 89% with *Sscrofa10.2* to 95% with *Sscrofa11.1*, the percentage of properly paired reads increased from 77 to 86%, and the percentage of reads with high mapping quality (MAPQ ≥ 40) increased from 71 to 84%. Here, we considered only SNPs but we expect that the alignment bias would have a greater impact when using a lower quality reference genome or in regions of high variability and structural complexity, e.g., in presence of multiple indels. Development of alternative-aware alignment algorithms or genome variation graphs [10, 33] could alleviate bias towards the reference genome due to alignment in the near future, but these methods still have some practical limitations and their use is not yet generalised.

### Index hopping

We estimated the level of index hopping in the 26 samples sequenced in a multiplex at 2× coverage to be equal to 1.5%. This was within expectations based on Illumina guidelines (< 2%) [16]. The impact of index hopping on the percentage of incorrect genotype calls depends on the conservativeness of the genotype calls. For conservative calls, the impact was negligible, but for best-guess genotype calls, the percentage of incorrect calls increased by 0.3 to 0.9 absolute percentage points (1.8–5.2% more incorrect calls).

We used a novel empirical method to estimate the level of index hopping that relies on sequencing the same set of samples twice, with and without multiplexing, such that the level of index hopping in the multiplexed data can be measured against a scale of simulated index hopping levels obtained from a set of index hopping-free data. Previously, Owens et al. [13] proposed a method for testing index hopping that was based on finding heterozygotes with unbalanced read counts for the reference and alternative alleles (e.g., one allele supported by many reads but the opposite allele only by one read), and then estimating index hopping based on the frequency of that opposite allele in the rest of individuals in the multiplex. The advantage of this method is that it uses existing data and does not require the same samples to be sequenced twice. However, this method requires high-coverage data and does not answer how index hopping affects genotype calls.

Our results, together with those of other studies [13, 14], reassure us that the high levels of index hopping reported by Sinha et al. [12] are unlikely to occur in most applications when good cleaning protocols are followed to remove excess free-floating indexing primers during library preparation or when unique dual indexes are used [15].

## Conclusions

Index hopping and bias towards the reference allele due to alignment have little impact on downstream genotype calls from low-coverage sequence data, but unexpected biases may arise from pipelines that use tools that were designed for high-coverage sequence data on low-coverage sequence data. The step of ‘pruning’ that is implemented in GATK HaplotypeCaller is an example of a feature that is desirable for high-coverage data but that introduces a systematic bias when applied to low-coverage data. We propose a simple new pipeline to correct this bias and we recommend that users of low-coverage sequence data be wary of unexpected biases before using bioinformatic tools that were designed for high-coverage sequencing.
